# Biocompatibility Computation of Muscle Cells on Polyhedral Oligomeric Silsesquioxane-Grafted Polyurethane Nanomatrix

**DOI:** 10.3390/nano11112966

**Published:** 2021-11-05

**Authors:** Touseef Amna, Mallick Shamshi Hassan, Mohamed H. El-Newehy, Tariq Alghamdi, Meera Moydeen Abdulhameed, Myung-Seob Khil

**Affiliations:** 1 Department of Biology, Albaha University, Albaha 65779, Saudi Arabia; msaa1258@gmail.com; 2 Department of Chemistry, Faculty of Science, Albaha University, Albaha 65779, Saudi Arabia; hassan4nano@gmail.com; 3 Department of Chemistry, College of Science, King Saud University, P.O. Box 2455, Riyadh 11451, Saudi Arabia; melnewhy@ksu.edu.sa (M.H.E.-N.); malhameed@ksu.edu.sa (M.M.A.); 4 Department of Organic Materials and Fiber Engineering, Jeonbuk National University, Jeonju 54896, Korea

**Keywords:** polyhedral oligomeric silsesquioxanes, polyurethane, muscle cells, nanofibers

## Abstract

This study was performed to appraise the biocompatibility of polyhedral oligomeric silsesquioxane (POSS)-grafted polyurethane (PU) nanocomposites as potential materials for muscle tissue renewal. POSS nanoparticles demonstrate effectual nucleation and cause noteworthy enhancement in mechanical and thermal steadiness as well as biocompatibility of resultant composites. Electrospun, well-aligned, POSS-grafted PU nanofibers were prepared. Physicochemical investigation was conducted using several experimental techniques, including scanning electron microscopy, energy dispersive X-ray spectroscopy, electron probe microanalysis, Fourier transform infrared spectroscopy, and X-ray diffraction pattern. Adding POSS molecules to PU did not influence the processability and morphology of the nanocomposite; however, we observed an obvious mean reduction in fiber diameter, which amplified specific areas of the POSS-grafted PU. Prospective biomedical uses of nanocomposite were also appraised for myoblast cell differentiation in vitro. Little is known about C2C12 cellular responses to PU, and there is no information regarding their interaction with POSS-grafted PU. The antimicrobial potential, anchorage, proliferation, communication, and differentiation of C2C12 on PU and POSS-grafted PU were investigated in this study. In conclusion, preliminary nanocomposites depicted superior cell adhesion due to the elevated free energy of POSS molecules and anti-inflammatory potential. These nanofibers were non-hazardous, and, as such, biomimetic scaffolds show high potential for cellular studies and muscle regeneration.

## 1. Introduction

Hybrid (organic/inorganic) nanocomposites prepared from polyhedral oligomeric silsesquioxane (POSS) have drawn substantial attention due to numerous beneficial purposes that typically originate from distinctive physical properties of POSS. Tissue engineering is a very promising field that offers surprising ventures in regenerative medicine. This area is focused on the search for substitutes to alleviate pain by reinstating the functions of injured cells and organs [1]. Therefore, the choice of material for designing scaffold remains a central objective for successful tissue engineering approaches [2]. Composite materials based on organic polymers and inorganic particles are employed for multiple purposes [3]. In this respect, POSS (possessing a cage structure of monomers) contributes as an edifice wedge for inorganic–organic fusion materials. Nevertheless, it has been observed that exchange of a POSS skeleton not only fetches variety but also formulates complementary and homogenously discrete POSS in organic polymers. It has also been observed that various properties of a number of inorganic–organic hybrid materials have been enhanced as compared to their nonhybrid equivalents, for instance, improved thermo-oxidative and ultraviolet steadiness [4], enhanced abrasion resistance [5] and hardness [6], and reduced flammability [7,8,9] and viscosity [10].

In this report, we portray the successful synthesis of a polyhedral oligomeric silsesquioxane (POSS)-grafted polyurethane (PU) blend of nanofibers via a facile electrospinning route. We chose the PU polymer and mouse myoblast cell lines (C2C12) for our study, because PU has marketable elastomers, which can allow for its use in adhesives, flexible films, coatings, and hard plastics [11,12], and C2C12 cells are a useful tool to explore mechanistic pathways [13]. When PU is supplemented with POSS, its features will advance; for instance, the mechanical strength, [14] thermo-oxidative stabilities [15,16], biodegradable resistance, [17,18] and fire resistance improve [19]. In their study, Zheng et al. utilized octa (aminophenyl)-POSS and octa(propyl glycidyl ether)-POSS as linkage materials for PU in order to create mechanical and thermal stability [20,21].

Keeping in consideration the distinctive characteristics of PU and POSS, we dispersed 10 wt.% of POSS molecules in a PU solution. A multipurpose and economical electrospinning method was utilized to spin the PU/POSS composite solution, and it is typically used to generate elongated and incessant fibers [22,23] for a variety of functions. As aforementioned, POSS is the most suitable material to be considered for hybrid fibers because of its finely organized compounds based on silica, which possesses a functional group on the outer surface [24]. Thus, we selected POSS molecules for constructing a blend scaffold. To the authors’ best knowledge, there are no reports in the literature in which the response of C2C12 mouse myoblasts has been inspected in relation to successfully designed POSS-grafted PU blend nanofibers. This research investigation is necessary to address the complexity of POSS-grafted PU blend nanofibers because of the unavailability of suitable donors or autografts. Nevertheless, the continuous screening of novel biomaterials in particular nanocomposites [25] is expected to be an innovative approach that will result in remarkable developments in tissue engineering.

## 2. Materials and Methods

### 2.1. Supplies

Polyurethane (PU, MW = 110,000, medical grade) was obtained from Cardio Tech. Intern., Tokyo, Japan. Isocyanatopropyl dimethyl silylcyclohexyl-polyhedral oligosilsesquioxane (POSS macromer) was a kind gift from Air Force Research Lab, Montgomery, OH, USA. Tetrahydrofuran (THF) and *N*,*N*-dimethylformamide (DMF) (analytical grade; Showa Chemicals Ltd., Tokyo, Japan) were utilized as solvents devoid of distillation. C2C12 cells and CCK-8 were obtained from American Type Culture Collection (ATCC) and Sigma–Aldrich Chemical Co., Burlington, MA, USA. Dulbecco’s modified Eagle medium (DMEM) was obtained from Gibco^®^ life technologies, Grand Island, NY, USA.

### 2.2. PU and POSS-Grafted PU Blend Scaffolds by Electrospinning

Fabrication of pure and POSS-loaded PU nanofibrous scaffolds were conceded using a simple electrospinning approach. PU (10 wt.%) was obtained through liquefying PU pellets in DMF: THF (1:1 *w*/*w*) with the help of magnetic shaking without heating. The POSS (10 wt.%) in THF was supplemented with PU (wt. ratio 10:1). The PU–POSS hybrid mixture was vigorously stimulated for 3 h without heating for appropriate assimilation. The PU–POSS hybrid mixture was transported into a 10 mL syringe to synthesize a POSS-grafted PU nanofibrous matrix. The electrospinning was conducted at a voltage of 12 kV and at a remoteness of 15 cm between the needle and collector. The pristine and blend nanofibrous scaffolds were dried in an oven at 60 °C for one day.

### 2.3. Characterization

Scanning electron microscopy (SEM: JEOL JSM5900, Tokyo, Japan) was applied to trace out the exterior morphology of the PU and POSS-grafted PU mats at different magnifications. The dried pristine (PU mat) and blend (POSS/PU) scaffolds were dispersed on carbon tape, layered with platinum by a sputter coater for 10 s at a current of 70 µA and a voltage of 15 kV. POSS-grafted PU nanofibers were screened for chemical concerto using an energy dispersive X-ray (EDX) spectrometer (JEOL JSM5900, Tokyo, Japan). The allocation of elements was meditated by electron probe microanalysis (EPMA). Fiber diameter was unswervingly considered from SEM metaphors. In all cases, 25 to 30 fibers from a randomly chosen section were calculated, and the mean was estimated. Fiber dimension distribution was measured using ImageJ (Scriptable Java app for scientific image processing) software. The XRD of PU and POSS-grafted PU blend nanofibrous scaffolds were documented on an X-ray diffractometer (D/MAX 2500, Rigaku Corporation, Tokyo, Japan) with copper Kα radiation (λ = 1.540 Å) over Bragg angles ranging from 10 to 60. The voltage and current of the apparatus were fixed at 30 kV and 40 mA, respectively. The effect on the polyurethane polymer structure after integration of POSS nanocrystals was examined by Fourier transforms infrared spectroscopy (FT-IR; Perkin Elmer, Walthan, MA, USA). The spectra of PU and POSS-grafted PU were verified by pulverizing respective mats with KBr to obtain pellets that were examined by adjusting the wave number from 4000 to 400 cm^−1^ at a resolution rate of 4 cm^−1^.

### 2.4. Cell–Scaffold Interaction Studies

The muscle cell–scaffold interactions on the designed POSS-grafted PU blend nanofibers were considered by culturing C2C12 in 75 cm^2^ culture flasks (Bedford, MA, USA). The C2C12 cells were revived in DMEM (pH 7.4) including 10% fetal bovine serum (FBS) and 1% penicillin–streptomycin. The cultivated C2C12 cells were sited in a humidified incubator and were provided with 5% CO_2_, 95% air, and a temperature of 37 °C.

The POSS-grafted PU nanofibrous scaffolds were cut into 1 × 1 cm dimensions, sterilized, and kept in the DMEM medium as described previously [26]. The POSS-grafted PU nanofibers were inoculated with C2C12 cells in 6-well microtiter culture plates for different incubation times. The profile of monocultured C2C12 was captured by scanning electron microscopy. Once proper confluent C2C12 cells emerged, cells were revitalized with phosphate-buffered saline (PBS) and then immobilized [26] and freeze dried as described elsewhere [27] for SEM examinations. The cell inoculated POSS-grafted PU nanofibers were air dried, Pt coated at a current of ~70 µA, and scanned for surface morphological features by Bio-SEM managed at a voltage of 10 kV. The C2C12 cells cultured in microtiter plates were utilized as a control.

The viability and vitality of C2C12 cells cultured on POSS-grafted PU scaffolds were scanned by using quantitative CCK-8 assay, which was performed spectrophotometrically. In brief, C2C12 cells were replenished with fresh media at 1, 3, and 5 days, and 10 μL of water-soluble tetrazolium-8 (CCK-8) was supplemented in individual wells and incubated. The results were obtained by considering absorbance at 450 nm by a spectrophotometer (model 680; Bio-Rad Laboratories, Hercules, CA, USA) as previously described [26].

### 2.5. C2C12 Differentiation on POSS-Grafted PU Nanoscaffolds 

C2C12 differentiation was studied on POSS-grafted PU nanoscaffolds as articulated earlier [28]. In brief, 5 × 10^4^ cells were cultured in culture dishes containing DMEM to gain 70–80% convergence. In due course, the exhausted media was reinstated with differentiation medium (DMEM with 5% (*v*/*v*) horse serum, 2 mM glutamine, penicillin (100 U/mL), and streptomycin (100 lg/mL)). The cultured myoblasts were maintained in differentiation medium until the termination of analysis (0–7 days). The formation of myotube was screened on a regular basis with a microscope.

### 2.6. Staining

The cultured myoblasts on POSS-grafted PU nanoscaffolds were bathed in phosphate-buffered saline, immobilized in absolute methanol approximately for 2–5 min, and subsequently air dried for 10 min. The Jenner staining (BDH, Poole, UK) was diluted as 1:3 (*v*/*v*) in 1 mM of sodium phosphate buffer (pH 5.6). Accordingly, 1 mL was dispensed among cultivated cells and incubated for about 5 min. The myoblasts were washed off using distilled water. Later, immobilized cells were marked with Giemsa stain (1 mL) prepared as 1:20 in 1 mM of sodium phosphate buffer and developed at room temperature for 10 min. Finally, C2C12 myoblasts were once more cleaned using DW. To end the process, the stained cells were captured from different angles with a camera equipped with an inverted microscope. The progress and expansion of myotubes were visualized using intense coloration with prominent nuclei. The unstained immobilized culture was sealed and stored at RT until future use.

### 2.7. Bactericidal Effect of POSS-Grafted PU Nanoscaffolds

In this study, *Staphylococcus aureus* (ATCC strain no.29213) was cultivated to evaluate antibacterial activity of POSS-grafted PU nanoscaffolds as previously reported [28]. The *S. aureus* culture was geared up on nutrient agar (Merck, Darmstadt, Germany). Individual colonies were picked up and preserved in cryovials at freezing temperature (−80 °C) in anticipation of further utilization. The *S. aureus* was grown (10^6^ CFU) with POSS-grafted PU nanoscaffolds (varying concentrations) to investigate the lowest amount needed to restrain biofilm formation. The established growth parameters were temperature: 37 °C, rpm: 150.

## 3. Results

The developments in tissue engineering have drawn incredible awareness to the field. The reason for this constantly growing demand is a result of its projected medical relevance for issues such as the renewal and rejuvenation of smashed tissues and organs. Currently, tissue engineering appears to be the most competent and noteworthy move that endeavors to present solutions for organ transplantation as well heralding innovative perspectives for management of diverse syndromes.

**Figure 1:**
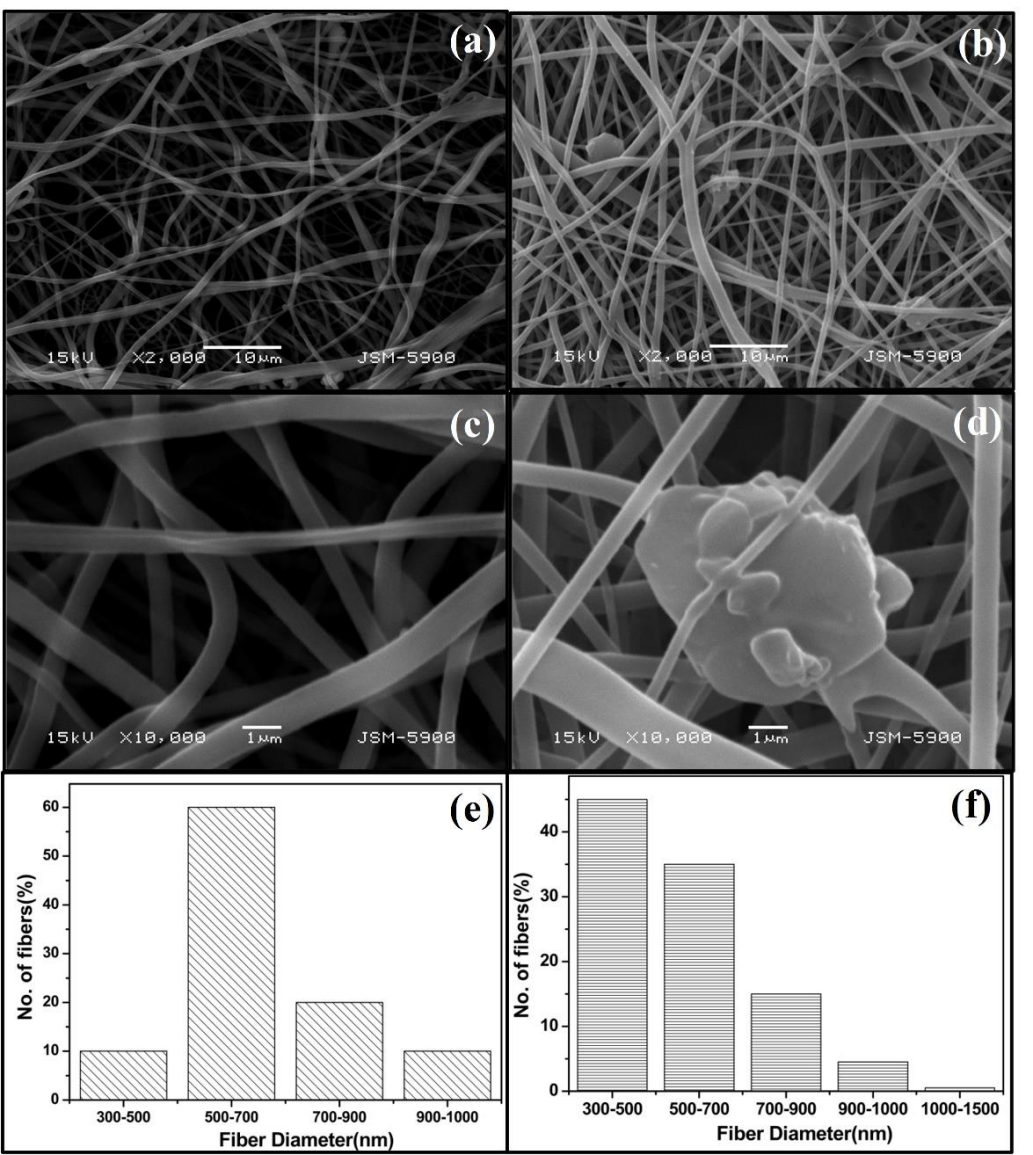
SEM metaphors of (**a**,**c**) pure PU and (**b**,**d**) POSS-grafted PU matrices at different intensifications and (**e**,**f**) fiber distribution of PU and POSS-grafted PU matrices, respectively.

**Scheme 1:**
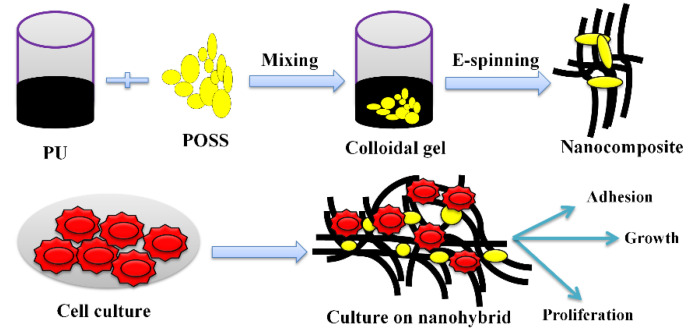
Schematic illustration of preparation of POSS-grafted PU nanomatrix and communication with muscle cells.

In this study, we optimized POSS concentration for successful preparation of nano-organic–inorganic hybrid (PU–POSS) scaffolds via a facile electrospinning route and investigated their response to C2C12 cells. Scheme 1 presents an orderly preparation of POSS-grafted PU nanomatrices created by an electrospinning process and their response to C2C12 cells. SEM imaging demonstrated synthesized PU and POSS-grafted PU nanofibers. SEM pictures of plain PU and POSS-grafted PU nanomatrices at different resolutions (low as well as broad) are shown in Figure 1. The pristine PU electrospun nanofibers had a comprehensible flat face without POSS molecules (Figure 1a,b). The POSS-grafted PU nanofibers had a comprehensible flat face bearing POSS molecules (Figure 1c,d). From SEM depiction, it was discovered that the integration of POSS into PU was even. POSS contains silica; thus, POSS-grafted PU blend nanofibers possess silica on their facades, and these silica particles were proved to be present, suggesting the potential of these nanofibers for biomedical purposes and, in particular, bone regeneration [24,29]. Moreover, it is a prerequisite in scaffold crafting that a scaffold should degrade automatically and progressively in order to allow fresh tissue formation. Thus, proscribed hydrolytic degradability and bio-resorbability of scaffolds in the body is crucial and, therefore, entails an advanced engineering move. Nevertheless, the focal point of biomedical engineering is material/biomaterial design in order to fulfill projected, ever-increasing medical demands. Therefore, the discovery and development of new and innovative biomaterials utilizing natural materials is a necessity [30]. In the present study, it was observed that electrospinning pristine PU resulted in fibers with an average diameter of 500–700 nm (±10 nm) (Figure 1a,c), while the PU polymer supplemented with a sufficient amount of POSS (10 wt.%) fashioned distinct nanofibers with an average diameter ranging from 300 to 500 nm (±10 nm) as seen in Figure 1b,d. A slight shrinkage in the diameter of POSS-grafted PU fibers was observed. Our results are consistent with previous reports. It is anticipated that the addition of POSS in PU promoted conductivity of the PU–POSS solution, and this elevated conductivity could be an outcome of the Si particles in POSS [31]. Additionally, POSS nanocrystals have been detected on PU-grafted POSS nanofibers. This confirms the blending of POSS in PU [32,33]. A decrease in the fiber diameter range was observed by loading POSS in PU fibers, which was further verified by numerical figures (Figure 1e,f). In the case of a PU–POSS nanocomposite, the average diameter shifted to a lower range (300–500 nm).

**Figure 2:**
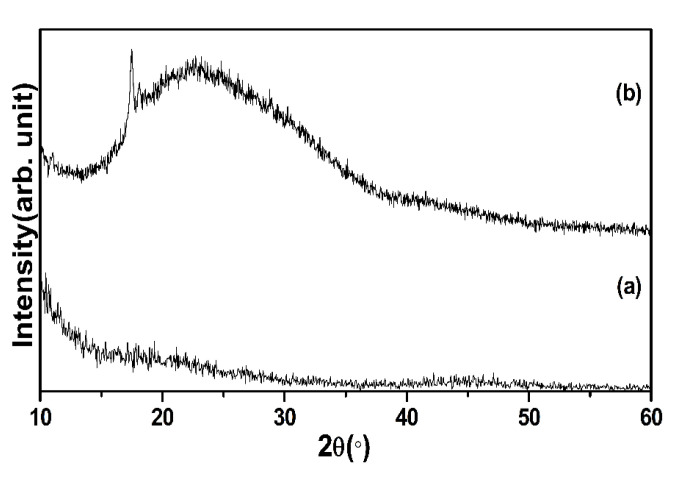
XRD spectra of pure (**a**) PU and (**b**) POSS-grafted PU nanomatrix.

**Figure 3:**
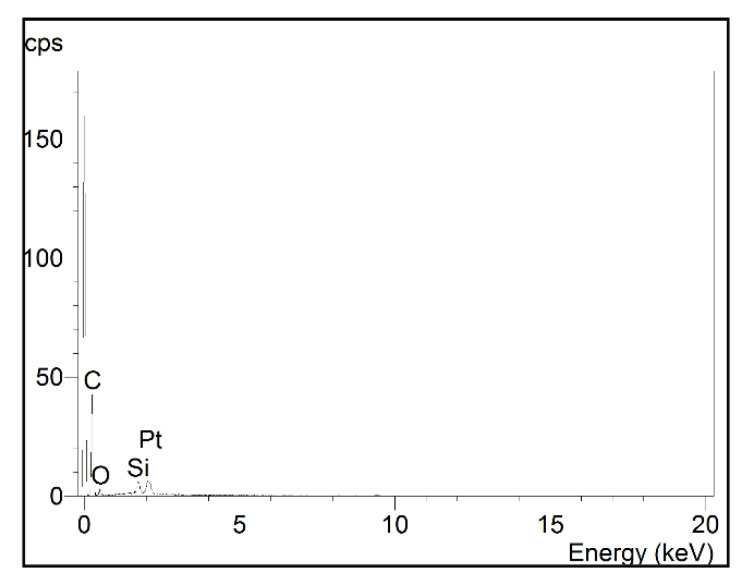
EDX spectrum of POSS-grafted PU nanomatrix.

The presence of dispersed POSS nanocrystals in hybrid matrices was also confirmed by XRD (Figure 2). Due to the amorphous behavior of PU polymer, no sharp structures were found in the XRD spectrum (Figure 2a) [34]. POSS-grafted PU nanofibers demonstrated a sharp peak at 18.0 degrees, which represents a crystalline POSS phase (Figure 2b). This suggests that POSS doping improved the crystallinity of the PU [35]. The successful blending of POSS nanocrystals with PU to yield POSS-grafted PU was also verified by EDX (Figure 3). In the EDX spectra of a POSS-grafted PU nanomatrix, carbon, oxygen, and silicon were detected without any contamination. The presence of Si was anticipated due to POSS nanocrystals.

**Figure 4:**
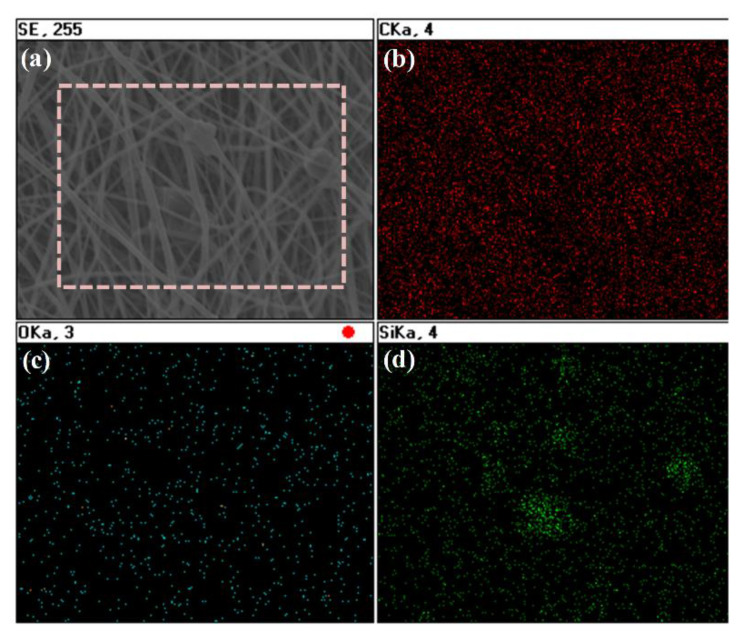
EPMA of (**a**) designated section of POSS-grafted PU nanomatrix demonstrating spatial distribution of elements (**b**) carbon, (**c**) oxygen, and (**d**) silicon.

Additionally, incorporation of POSS into PU was also established by EPMA (Figure 4). In the EPMA micrograph, carbon was found as a key component, whereas Si and O were homogeneously dispersed.

**Figure 5:**
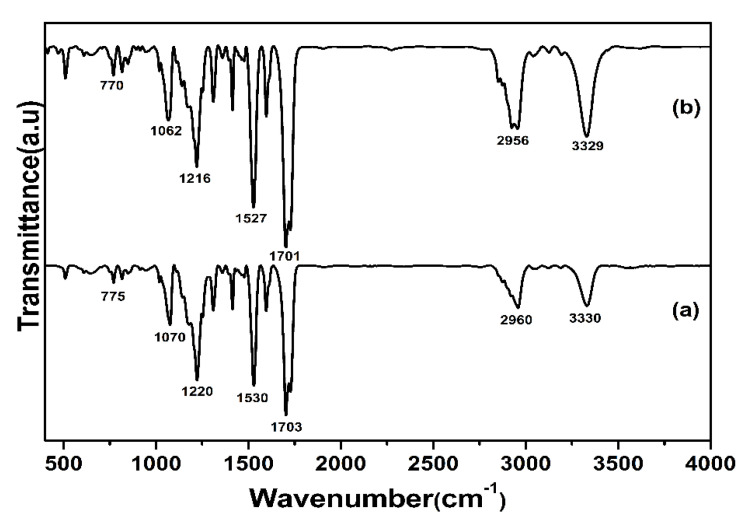
FT-IR spectra of (**a**) pure PU and (**b**) POSS−grafted PU nanomatrix.

To further investigate the interaction between POSS nanocrystals an PU, FT-IR analysis was performed. The infrared spectrum of PU and POSS-grafted PU is illustrated in Figure 5. The FT-IR of PU has characteristic assimilation bands at 3330, 2960, 1703, 1530, 1220, 1070, and 775 cm^−1^, which are assigned to *ν*(N–H), *ν*(C–H), *ν*(C=O), *ν*(C=C), *ν*(C–C), *ν*(C–O), and *ν*(C–H), respectively, on substituted benzene [36,37] (Figure 5a). In POSS-grafted PU nanofibers, there were no pronounced change in FT-IR when matched up to the pure PU. However, by loading POSS particles in PU, the peak intensity increased (Figure 5b). The increase in intensity on the hybrid spectrum shows that POSS was homogeneously distributed throughout the PU matrix. Most of these vibrations represent small changes, and little shifting was observed at all bands.

**Figure 6:**
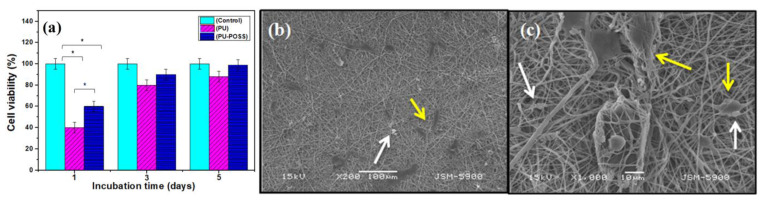
Cell counting assay-8 data (**a**) variability of control was positioned at 100%, and viability reference to control was articulated. At day one of incubation, significant differences (* *p* ≤ 0.05) in cellular response were observed on PU and POSS-grafted PU. However, a less significant difference was observed with an increase in time on both the nanomatrices with respect to the untreated control. The experiment was performed at least in triplicates, and divergence was considered statistically significant at a value of * *p* ≤ 0.05. Statistical computations were run on SPSS statistical software by an unpaired Student’s *t*-test. Illustrative SEM images (**b**,**c**) point to propagation on POSS-grafted PU nanomatrix. Yellow arrows point to myoblasts and white arrows POSS molecules.

After optimizing the weight percentage of POSS in the PU solution and the successful fabrication of POSS-grafted PU scaffold, cell–nanofiber interactions of pure PU and POSS-grafted PU scaffolds in this study were further investigated. The POSS-grafted PU scaffolds were scrutinized for biocompatibility with C2C12 cells. The C2C12 cells were seeded on PU and POSS-grafted PU scaffolds, and, correspondingly, biocompatibility assay was carried out for one, two, and three days. Concerning the POSS-grafted PU nanomatrix, the quantum of healthy cells exponentially amplified. In the case of PU, no toxicity was found, but a higher number of live cells were on POSS-grafted PU nanofibers (Figure 6a). It was noted that, in the initial period of growth, attachment and propagation were more advanced on the POSS-grafted PU nanomatrix, as pendant nanocage enclosing silicon atoms may have outlined the foci of silicon-rich regions with improved surface-free energy facilitating endothelialization and deterred coagulant proteins, as previously reported [38]. The morphology of cells on POSS-grafted PU scaffolds was assessed using Bio-SEM (Figure 6b,c). Specifically, electrospun POSS-grafted PU nanofibrous scaffolds showed higher initial cell attachment and proliferation, which may be because of the elevated surface-free energy of POSS siloxanes [29].

**Figure 7:**
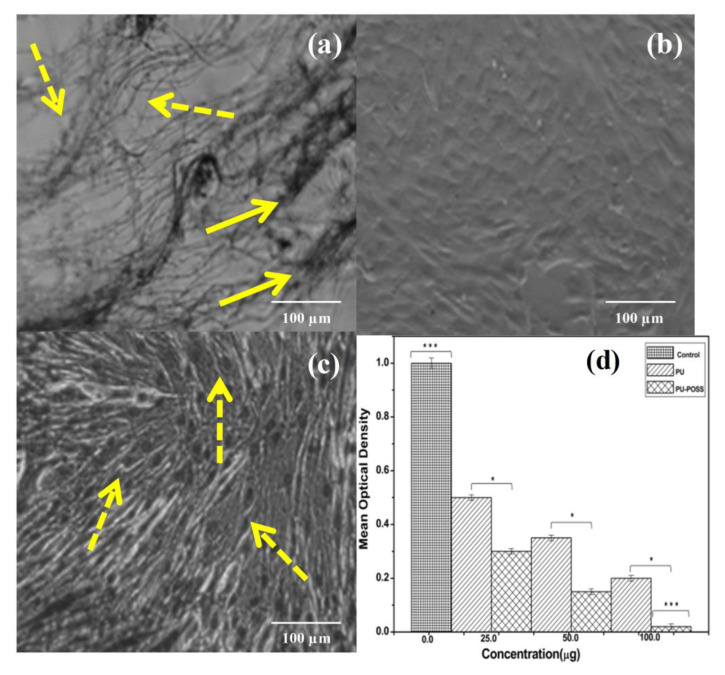
Microscopic metaphors of (**a**) growing myoblasts on POSS-grafted PU nanomatrix. Dashed bright yellow arrows point to POSS molecules, whereas solid arrows indicate spreading of myoblasts (**b**) confluent growth of C2C12 cells just prior to differentiation (**c**) Giemsa tinge depiction of myotube creation with prominent nuclei (dashed yellow arrows) during differentiation in petite quantity of serum. Bar graph illustration of (**d**) OD (600 nm) of spent culture supplemented with varying amounts of PU and POSS-grafted PU (100% expansion symbolize *S. aureus* in culture medium without nanomatrices). Significant difference (* *p* ≤ 0.05) was observed in the inhibition of bacteria between the control group and treatments at all concentrations. In addition, there is a significant difference in the bacteriostatic effect on PU and POSS-grafted PU nanomatrix at a high concentration. * *p* ≤ 0.05, *** *p* ≤ 0.001 significantly different from inoculated control.

Additionally, a simplistic way to estimate myotube development was designed in this study. Figure 7a–c illustrate randomly taken images and an estimation of intriguingly marked nuclei, which were employed to determine myotube compactness. Interestingly, the POSS-grafted PU scaffolds confirmed antibacterial potential. *S. aureus* depicted exponential doubling during the timeframe between 4 to 8 h in a virgin environment (Figure 7d). The entire period to monitor the influence of POSS-grafted PU scaffolds was set to 16 h, and an outstanding reticence of bacterial cultures was observed at a high concentration. Earlier studies have also discussed the formation of POSS-based nanocomposites and bilayered scaffolds, which have demonstrated excellent antibacterial and anti-inflammatory characteristics in addition to inducing cell growth (Scheme 1). Admittedly, our study is in agreement with various previous reports [39,40].

## 4. Conclusions

In conclusion, nanoscaffolds with superior biocompatibility as well as proper potency to retain their form can be used for biointegration and will unquestionably provide a priceless substitute to current materials. Our study highlights the benefits of POSS grafting for C2C12 cellular biocompatibility. The POSS-grafted PU scaffolds were fabricated by electrospinning. The SEM-EDX, EPMA, and XRD analyses clearly confirmed the formation of a POSS-grafted PU nanoscaffold. In the present study, it was established that the addition of POSS in PU-shaped grafted fibers had a smaller width and lesser diameter distribution than those of electrospun PU nanofibrous scaffolds. Furthermore, it was noted that cell interaction and dispersal behavior were more expressive in the case of POSS-grafted PU nanofibers. We hypothesize that POSS-grafted PU scaffolds possess a superior surface-free energy of POSS siloxanes; therefore, they are responsible for fine attachment and proliferation. The POSS-grafted PU nanoscaffold will act as a novel contrivance for nanomedicine, tissue engineering, and C2C12 differentiation.

## Data Availability

Not applicable.
